# Advances in Proteomics Allow Insights Into Neuronal Proteomes

**DOI:** 10.3389/fnmol.2021.647451

**Published:** 2021-04-15

**Authors:** Erin Fingleton, Yan Li, Katherine W. Roche

**Affiliations:** National Institute of Neurological Disorders and Stroke (NINDS), Bethesda, MD, United States

**Keywords:** neuroproteomics, schizophrenia, autism spectrum disorder, BONCAT, SILAC, mass spectrometry, proximity ligation assay, Alzheimer’s disease

## Abstract

Protein–protein interaction networks and signaling complexes are essential for normal brain function and are often dysregulated in neurological disorders. Nevertheless, unraveling neuron- and synapse-specific proteins interaction networks has remained a technical challenge. New techniques, however, have allowed for high-resolution and high-throughput analyses, enabling quantification and characterization of various neuronal protein populations. Over the last decade, mass spectrometry (MS) has surfaced as the primary method for analyzing multiple protein samples in tandem, allowing for the precise quantification of proteomic data. Moreover, the development of sophisticated protein-labeling techniques has given MS a high temporal and spatial resolution, facilitating the analysis of various neuronal substructures, cell types, and subcellular compartments. Recent studies have leveraged these novel techniques to reveal the proteomic underpinnings of well-characterized neuronal processes, such as axon guidance, long-term potentiation, and homeostatic plasticity. Translational MS studies have facilitated a better understanding of complex neurological disorders, such as Alzheimer’s disease (AD), Schizophrenia (SCZ), and Autism Spectrum Disorder (ASD). Proteomic investigation of these diseases has not only given researchers new insight into disease mechanisms but has also been used to validate disease models and identify new targets for research.

## Introduction

**FIGURE 1:**
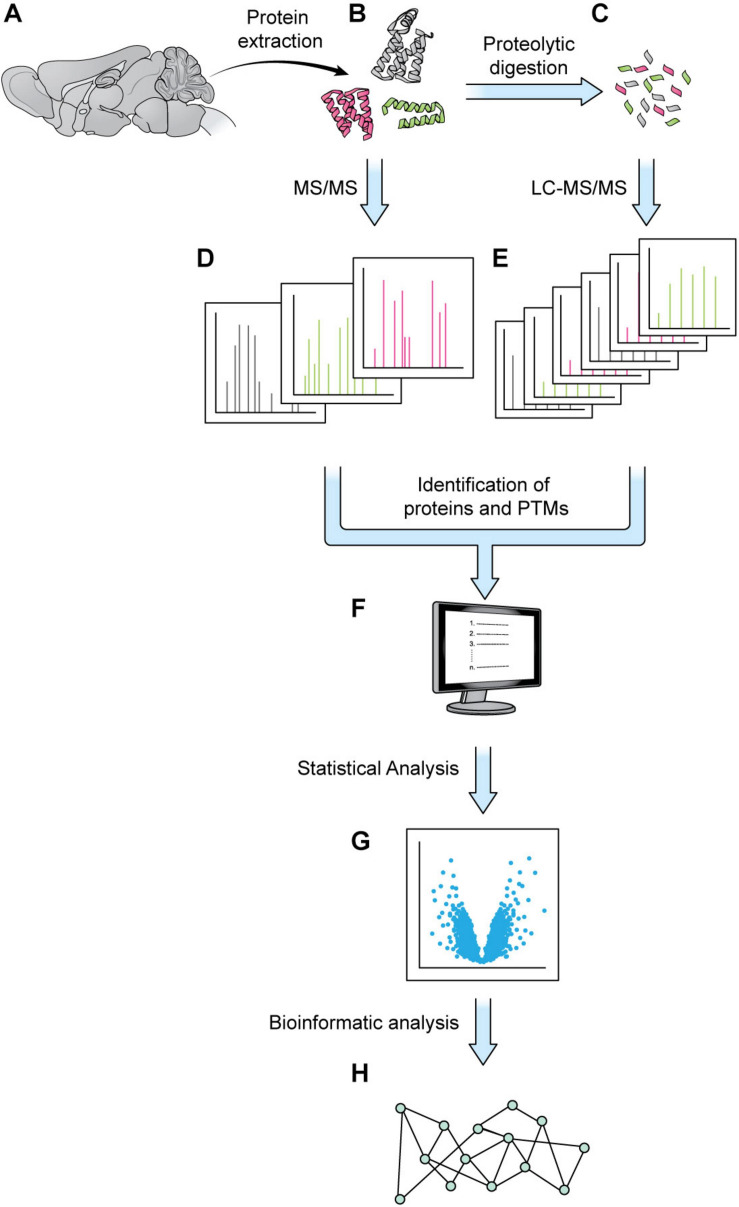
Generic mass spectrometry-based proteomics workflow. Proteins are extracted from a biological sample. With the bottom-up approach, proteins are digested into shorter peptides, then used for LC-MS/MS data acquisition (steps **A–C**,**E**, without step **D**). With the top-down approach, the intact protein is subjected to MS/MS analysis without digestion (steps **A**,**B**,**D**, no steps **C,E**). MS1 and MS2 data are processed by a database search or a *de novo* sequencing algorithm to obtain protein sequence and PTM status **(F)**. The abundance of MS1 and/or MSn signal can be employed for protein quantitation and statistical analysis **(G)**. Various bioinformatics tools such as GO analysis or pathway analysis can be used to extract biologically relevant information from proteins of interest discovered in proteomics analysis **(H)**.

Elaborate and tightly regulated protein–protein interaction (PPI) networks underlie key neuronal processes like axon guidance and synaptic plasticity, which are essential for the initial wiring and ongoing plasticity of the brain. Every neuron contains a multitude of distinct PPI networks, which must be highly compartmentalized to permit efficient transmission and encoding of information. This proteomic intricacy is compounded by the various cell-types and substructures that make up the brain. Additionally, these networks must be dynamic and flexible to allow the neuron to respond to new patterns of information and changing extracellular environments. This level of complexity presents a major stumbling block for scientists trying to understand the brain through traditional biochemical techniques, which are limited in efficiency of throughput and level of resolution. Over the last few decades, mass spectrometry (MS) has gained popularity as a powerful tool for quantifying and identifying the constituent peptides of any proteome. Traditionally, weeks of MS analysis were required to fully catalog and identify even simple proteomes, such as baker’s yeast (Bekker-Jensen et al., 2017). Modern techniques are not only faster but can achieve a proteome coverage comparable to that of RNA-seq, while additionally granting insight to post-translational modifications (PTMs) (Bekker-Jensen et al., 2017). Combined with modern protein-labeling techniques, MS can also be used to identify and quantify cell-specific, subcellular, and temporally dynamic proteomes, filling important gaps that are not addressable using genomic and transcriptomic techniques. The details of MS are well-reviewed elsewhere, but generally most MS experiments can be viewed as comprising three steps: (1) sample preparation, (2) MS data acquisition, and (3) data analysis and interpretation (Liao et al., 2009; Aebersold and Mann, 2016; Hosp and Mann, 2017; Figure 1).

**FIGURE 2:**
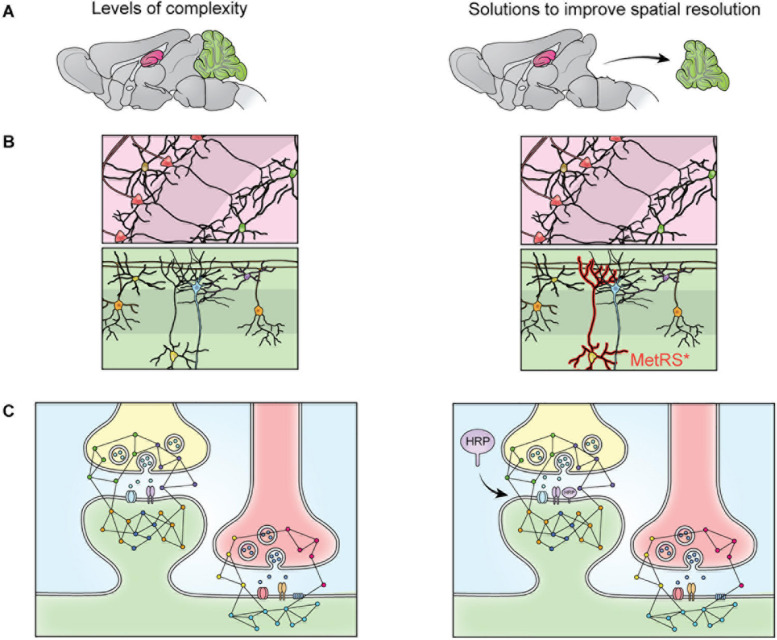
The heterogeneity of the nervous system prevents isolation of specific proteomes though fractionation. The brain contains many substructures, such as the hippocampus (pink) and cerebellum (green) **(A)**. When the brain is homogenized, the proteomes of these structures are mixed and no longer distinguishable by MS. This limitation can be overcome via dissection **(A)**, but cellular heterogeneity remains **(B)**. When dissected brain areas are homogenized, the proteomes of specific cell types become indistinguishable via MS, this can be overcome by cell-specific tagging of proteins. For example, mutant methionine transferase (MetRS*) can be expressed in a cell specific manner to permit tagging and purification of proteins with a bio-orthogonal amino acid **(B)**. Cells of the nervous system are also highly compartmentalized. Specific cellular compartments, such as the synaptosome, can be isolated via fractionation, but isolation of specific synapse types is not possible with fractionation **(C)**. This can be addressed by targeting a protein labeling enzyme (here, HRP) to a specific subcellular locale, where it will label only nearby proteins **(C)**.

**FIGURE 3:**
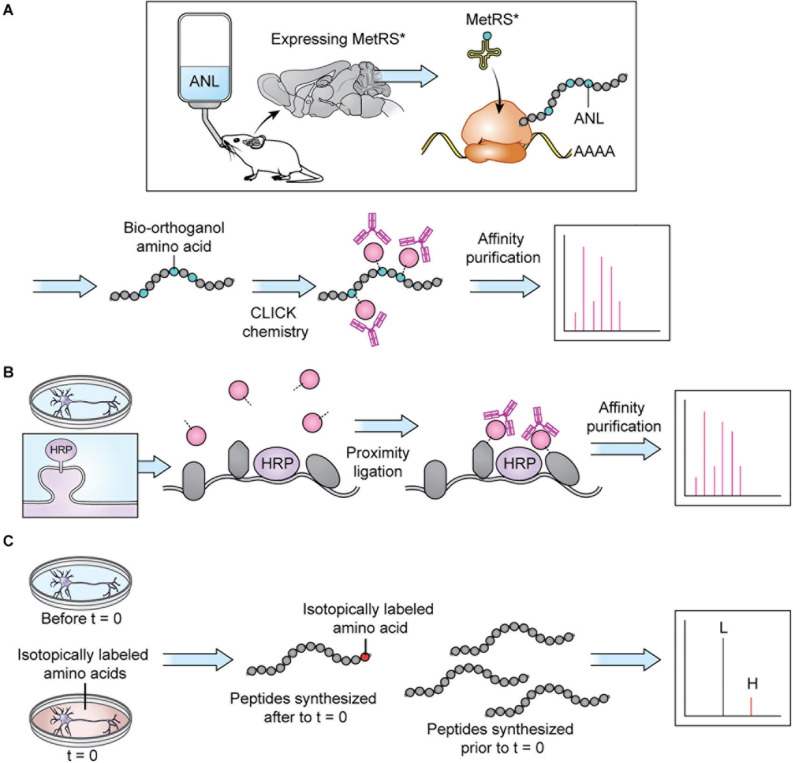
Protein tagging techniques allow MS analysis of specific proteomes. **(A)** BONCAT: a modifiable bio-orthogonal amino acid (here: ANL) is incorporated into a newly synthesized protein via a mutant methionine transferase. During sample preparation, the bio-orthogonal amino acid is modified via CLICK chemistry to permit addition of a small molecule permitting affinity purification prior to MS. Stochastic orthogonal recoding of translation (SORT) operates by similar principles. For *in vivo* applications, the bio-orthogonal amino acid can be introduced in the animal’s diet. Panel adapted from Alvarez-Castelao et al. (2017). **(B)** Proximity ligation: An enzyme (here: HRP) is targeted to a specific cellular locale where it catalyzes reactions allowing small molecule tagging of nearby proteins. Tagged proteins are then affinity purified before MS. In cell culture applications, the small molecule can be introduced in the cell culture media. Panel adapted from Loh et al. (2016). **(C)** SILAC: an isotopically labeled amino acid is incorporated into a newly synthesized protein, causing a shift in mass visible on m/z spectra, allowing spectra of newly synthesized peptides (H) to be distinguished from spectra of peptides synthesized prior to delivery of the isotopically labeled amino acids (L). In cell culture applications, the isotopically labeled amino acids can be introduced in the cell culture media.

Sample preparation has become more sophisticated over the last decade and varies depending on the specific goals of the research. Given the large dynamic range of protein concentration and the low abundance of certain subcellular proteomes, thorough proteome screening and PTM profiling often require techniques to enrich specific protein populations. Besides increasing the sensitivity of the MS analysis, enrichment coupled with highly sensitive and accurate MS instruments also allows for more targeted datasets (Kitchen et al., 2014). To characterize interactomes, researchers can affinity purify a protein of interest prior to MS. Researchers interested in understanding PPI networks more broadly can use crosslinking MS to gain insight into how proteins interact within a proteome without restricting analysis to a specific protein’s interactome (Gonzalez-Lozano et al., 2020). Other approaches do not offer insight into PPIs but allow purification of spatially and temporally restricted proteomes. Spatial resolution can be introduced at the brain region level though dissection and, more recently microdissection (MacDonald et al., 2019; Figure 2A). To achieve subcellular spatial resolution, researchers have traditionally relied on fractionation, which is a powerful technique for enriching organellar and synaptosomal proteomes (Biesemann et al., 2014; Itzhak et al., 2016, 2017). However, these techniques cannot specifically enrich neuronal proteomes over glial proteomes or excitatory synaptic proteomes over inhibitory synaptic proteomes, and is incapable of enriching some proteomes at all, at times rendering MS a blunt tool (Loh et al., 2016; Hosp and Mann, 2017; Figure 2). This shortcoming can be partially addressed through the use of cell sorting techniques, such as fluorescence activated cell sorting (FACS) and magnetic activated cell sorting (MACS), which can separate cellular material based on cell-type specific expression of a fluorophore or cell-type specific labeling with magnetic beads prior to analysis with MS. These techniques have been used to successfully purify nuclei, cell bodies, and synaptosomes from neurons (Biesemann et al., 2014; Poulopoulos et al., 2019). However, cell sorting approaches involve the dissociation and resuspension of tissues, which may shear the long processes characteristic of neurons, astrocytes, and other glial cell types, preventing these proteomes from being captured by cell sorting. Cell-type specificity can also be introduced, to some extent, *in vitro* through the use of cell culture protocols that enrich specific cell-types. Researchers looking for *in vivo* solutions capable of resolving the entire proteome of specific cell-types or otherwise unreachable subcellular proteomes, can turn to protein labeling techniques such as Bio-orthogonal non-canonical amino acid tagging (BONCAT), stochastic orthogonal recoding of translation (SORT), and Ascorbate Peroxidase (APEX) Labeling, which involve genetic expression of an enzyme or tRNA that facilitate the tagging of proteins (Dieterich et al., 2006; Roux et al., 2013; Elliott et al., 2014, 2016; Lobingier et al., 2017; Branon et al., 2018; Krogager et al., 2018; Figures 2B, 3B,C). Due to the genetic nature of these approaches, they can be combined with conditional expression systems, such as Cre-loxP, to grant cell specificity (Figure 2B). Similarly, approaches involving tagging an organelle of interest can be combined with Cre-loxP to permit purification of specific organelles prior to MS (Fecher et al., 2019). Additionally, approaches that involve enzymatic modification of a protein, like APEX, can be used to grant further spatial resolution by targeting the modifying enzyme to a specific cellular locale, and thus only labeling proteins in that vicinity (Figures 2C, 3B). These techniques, along with stable-isotope labeling with amino acids in cell culture (SILAC), involve introduction of an exogenous factor–either a modified amino acid or ligand–that labels the protein (Ong et al., 2002). By using pulse-chase approaches to the application of these exogenous factors, researchers can introduce temporal resolution to their studies. SILAC in particular has been used to great effect to study the role of protein turnover in neurons (Savas et al., 2012; Dörrbaum et al., 2018; Fornasiero et al., 2018; Heo et al., 2018). Coupled with sophisticated protein tagging techniques, MS can be used to study specific subsets of the proteome with considerable spatial and temporal resolution (Liao et al., 2009; Yuet and Tirrell, 2014; Hosp and Mann, 2017; Figure 3). Additionally, the development of techniques to enrich peptides with PTMs and sufficiently sensitive and accurate MS instruments affords researchers an exquisite level of insight into PTM-mediated signaling pathways.

Once proteins are extracted from a biological sample, often one of two principal approaches are employed during MS analysis: the conventional bottom-up or the less commonly used top-down method (Mann and Wilm, 1994; Yates et al., 1995; Kelleher et al., 1999). When the bottom-up method is used, proteins are digested into shorter peptides, then analyzed by liquid chromatography tandem mass spectrometry (LC-MS/MS). Whereas during a top-down analysis, the intact protein is isolated and subjected to MS/MS fragmentation without digestion. For both approaches, the survey scan (MS1) measures the molecular weight of peptides/proteins; during the product ion scan (MS2), peptides/proteins are dissociated with various fragmentation methods, and peptide/protein fragments are acquired. The pattern of the product ions is determined by the amino acid sequence of the specific peptide/protein. MS1 and MS2 data are processed through a database search or a *de novo* sequencing algorithm (Eng et al., 1994; Devabhaktuni and Elias, 2016). Protein sequence can thus be inferred directly (in top-down workflow) or via peptides matched to the protein (in bottom-up workflow). PTMs can be identified as well, combining the information in MS1 and MS2 data. Product ions can be further fragmented (MSn) to more specifically determine protein sequence, detect specific modifications occurred, and obtain more accurate quantification (Schey et al., 1989). For quantitative analysis, label-free quantitation (LFQ) method directly compares precursor ion abundances of peptides among different LC-MS/MS runs. For label-based approaches, such as Isobaric Tags for Relative and Absolute Quantification (iTRAQ) and tandem mass tags (TMT), the N-terminus and the lysine side chain of peptides are isotopically labeled. After labeling, different samples are mixed and subjected to LC-MS/MS experiment. The abundance of MS2 or MSn reporter ions is used to quantify different samples. Another commonly used labeling methods is SILAC, in which a combination of 13C and 15N labeled Lysine and Arginine is incorporated into proteins by growing cells in “heavy” medium, resulting in a mass shift of peptides at MS1 level once the protein is digested with trypsin, allowing differentiation of peptides containing heavy isotopes from peptides with medium isotopes or without labeling (Ong et al., 2002; Schwanhäusser et al., 2009; Cagnetta et al., 2018).

In the past decade, advances in mass spectrometers include not only the improvement in mass accuracy and resolution, sensitivity, and scan speed, but also the development of robust fragmentation methods (Yates, 2019). With the increase in the power of MS, the list of protein hits returned from any experiment is much longer than those of early MS experiments, and demands a modern approach to analysis and interpretation. To address this need, researchers have developed bioinformatic approaches capable of grappling with the complexity and richness of MS-generated proteomic datasets, producing easily interpretable birds-eye views of proteomic data (Liao et al., 2009; Ghiassian et al., 2015). There is a variety of proprietary and open-access data analysis tools available to the modern researcher and covering all of them is beyond the scope of this review. They are well reviewed elsewhere (Wu et al., 2014; Chen et al., 2020). Here we will briefly describe the concepts underlying popular MS analysis approaches. Much of bioinformatic analysis relies on making inferences about a MS dataset based on what is already known about protein function. As such, bioinformatic analyses typically involve labeling each identified protein with its associated gene ontology (GO) term. GO terms can reflect the protein’s biological function, cellular localization, biological pathway, or disease association, and can be sourced from several databases, such as GoMiner, KEGG, or the more recent SynGO. Based on GO term assignation, researchers can statistically identify GO terms that are enriched in their dataset–or appear more frequently than expected by chance–to gain insight into which biological processes, pathways, and locales might be reflected in their proteomic dataset. Additionally, software packages like Qiagen’s Ingenuity Pathway Analysis tool, Thermo fisher’s ProteinCenter, or the open-access Pathway Commons can be used to visualize the PPI networks and pathways represented in the MS dataset. Continuing advances made in areas from cell-specific purification, protein labeling, enrichment of peptides with PTM, MS instrumentation, data interpretation and bioinformatic analysis have elevated MS-based proteomics analysis from a general tool to a precision instrument with new relevance to research in both basic neuroscience and neurological disease.

## MS in Basic Biology

To complete the computational work of the brain, neurons rely on very fast and highly compartmentalized signaling processes, and are supported by an array of glial cells, each with their own subcellular compartments and signaling pathways. Detailed proteomic snapshots of these cells are central to understanding the contours of how these signaling events unfold and the cellular processes they underlie. The power of MS in approaching questions in basic biology is demonstrated by research on the synaptic vesicle (SV) proteome. Using fractionation to purify SVs prior to MS, Takamori et al. (2006) were able to identify and quantify the proteomic content of the average SV, revealing important insights into how SVs are likely to function. Since then, significant advances in MS and protein enrichment techniques have opened access to previously unreachable proteomes, which will be the focus of this section. We will begin with efforts to target the proteomes of specific neuronal cell-types and subcellular domains. We will then review the use of stable isotope labeling in studies on newly synthesized peptides during axon guidance. Finally, we will discuss the use of phosphoproteomics in understanding signaling events in long-term potentiation (LTP). Interestingly, each of these subjects are well-studied, yet MS studies are still capable of unveiling new molecular players and pathways, providing researchers with new avenues for investigation.

### Subcellular and Cell-Type Specific Mapping Efforts

As a cell-specific enrichment approach, BONCAT relies on the integration of a modifiable non-canonical amino acid into newly translated proteins (Dieterich et al., 2006; Figure 3A). Integration of the non-canonical amino acid is only possible with expression of a mutant methionine transferase (MetRS^∗^). When MetRS^∗^ expression is coupled with a cell-specific Cre-loxP system, proteins are tagged with the non-canonical amino acid in a cell-specific manner. Subsequent CLICK chemistry modification of the non-canonical amino acid allows purification of proteins prior to MS (Dieterich et al., 2006; Alvarez-Castelao et al., 2017). Alvarez-Castelao et al. (2017) implemented BONCAT in a comparison of hippocampal excitatory and cerebellar inhibitory neurons. Interestingly, they found the MS profiles of dissected hippocampus and cerebellum to be more similar to glial MS profiles than the MS profiles of each brain area’s major constituent neuronal cell type (CaMKII+ and GAD2+, respectively), suggesting that the glial proteome contributes significantly to MS datasets derived from dissected tissues. They also identified a number of significantly differentially expressed proteins in CaMKII+ and GAD2+ neurons. While some of their findings are unsurprising–PSD-95 is enriched in CaMKII+ neurons, and calbindin is enriched in GAD2+ neurons–their results also identify some unexpected proteins and lend insight into which cellular pathways are important for these two highly studied cell types. Although this study focused on adult tissue, application of cell type-specific BONCAT in younger animals may shed a light on how different cells in the nervous system initially develop and wire up with their neighbors.

Researchers can see beyond cellular proteomes and zero-in on specific subcellular locales using proximity labeling assays, like APEX, BioID, and TurboID (Roux et al., 2013; Lobingier et al., 2017; Branon et al., 2018; Figure 3B). These approaches involve targeting an enzyme to an area of interest where it can modify a freely diffusing molecule–usually biotin–which, once modified, can attach itself to nearby proteins, allowing their later purification. It is important to note that, much like BONCAT, this approach requires expression of an exogenous enzyme and as such requires use of a transfection method or development of a knock-in mouse line. Loh et al. (2016) targeted horseradish peroxidase (HRP) to inhibitory and excitatory synapses, allowing purification of excitatory and inhibitory synaptic proteins with high specificity. Despite the relatively high focus these subcellular areas have received, Loh et al. (2016) were able to identify several synapse and synapse-type orphans: proteins that were not previously known to be synaptic or specific to a type of synapse, respectively. Intriguingly, they identify MDGA1 as an excitatory synaptic protein, which was unexpected due to its high sequence homology to MDGA2, which is inhibitory, and the fact that both have been shown to bind to neuroligin-2, an inhibitory synaptic adhesion molecule. As a group, neuroligins are synaptogenic. In overexpression paradigms, neuroligin-2 can stimulate excitatory synaptogenesis, despite its identity as an inhibitory neuroligin. Loh et al. (2016) go on to demonstrate that MDGA1 may be responsible for dampening neuroligin-2 mediated excitatory synaptogenesis, suggesting that in overexpression paradigms neuroligin-2 can overwhelm endogenous MDGA1, thus permitting excitatory synaptogenesis. Further study of MDGA1 and MDGA2 will likely lead to a new understanding of how synapse specificity arises and is maintained.

### Axon Guidance

Axon guidance, the molecular signaling mechanism that allows axons to find their appropriate post-synaptic partners, is crucial for brain patterning and the development of neural circuits. As the axon extends, receptors on the growth cone, a fan shaped protrusion at the end of the axon, respond to attractive or repulsive guidance cues and initiate signaling cascades that remodel the cytoskeleton of the growth cone accordingly (Chilton, 2006; Bellon and Mann, 2018). A multitude of axon guidance receptors and cues exist, but all are generally thought to converge on the same cytoskeleton remodeling pathways, allowing integration of multiple signals which ultimately guide the axon toward attractive cues and away from repulsive cues (Chilton, 2006; Bellon and Mann, 2018). Local translation is key to this effort, necessitated by the fact that the axon guidance occurs at great distances from the soma, where most protein synthesis occurs (Spaulding and Burgess, 2017). Although axonal transcription is known to be crucial to axon guidance, how different guidance cues sculpt the transcriptional landscape is not well defined.

In their 2018 Neuron paper, Cagnetta et al. (2018) set out to answer this question using pulsed SILAC (pSILAC), an approach in which labeled lysine and arginine are incorporated into newly synthesized proteins of different cell states (Ong et al., 2002; Schwanhäusser et al., 2009; Figure 3C). The authors exposed isolated *Xenopus laevis* retinal ganglion cell (RGC) axons to three attractive guidance cues–netrin-1, Sema3a, and BDNF–paired with isotopically labeled amino acids and analyzed the resulting proteome via MS, thereby identifying the translational changes induced by each guidance cue. Their results indicate that while all three guidance cues cause the same translational changes in a subset of proteins–supporting the idea that guidance cues generally converge on the same pathways–each guidance cue also initiates a unique program of translational changes, which might grant insight into why several guidance cues are needed to signal what essentially boils down to “come here” vs. “go away.”

Some guidance cues are attractive in one context, but repulsive in another (Chilton, 2006). Cagnetta et al. (2018) identified the translational changes induced by the same guidance cues in repulsive circumstances. Nearly 75% of newly synthesized proteins underwent the opposite translational change (i.e., if translation was down-regulated during the attractive cue, it was up-regulated during the repulsive cue and vice versa), supporting the idea that repulsive and attractive cues converge on the same pathways in opposite manners. The remaining ∼25% that undergo the same translational change regardless of the valence of the cue point to conserved aspects of attractive and repulsive axon guidance. In a later study, proteomic analysis of the guidance cue receptor interactomes revealed that different receptors interact with specific ribosomes, mRNAs, and mRNA binding proteins, which may explain the unique translational signature of each guidance cue (Koppers et al., 2019). These studies add to a rich and growing body of literature tackling the role of *de novo* peptide synthesis and axonal protein transport in axon guidance across model organisms (Poulopoulos et al., 2019; Schiapparelli et al., 2019).

### Synaptic Plasticity

The incredible plasticity of the brain allows us to learn and consolidate knowledge in an ever-changing environment. There are many forms of synaptic plasticity, including LTP, long-term depression (LTD), and homeostatic plasticity, such as synaptic scaling (SS). LTP is the process by which synapses undergo an increase in synaptic efficacy after a period of high-frequency activity, whereas LTD is the loss of synaptic efficacy after a period of low-frequency activity. SS keeps LTP and LTD in check by dampening or strengthening synapses after long periods of high activity or low activity, respectively, which prevents synapses from undergoing LTP or LTD ad infinitum and maintains the dynamic range of synapses. These forms of plasticity are widely thought to be the molecular correlates of learning and memory, and appear key to the maintenance and tuning of neural networks.

NMDA receptors (NMDAR) play a central role in many forms of synaptic plasticity. They are a distinct class of Glutamate receptors that act as coincidence detectors by being activated upon depolarization and ligand binding. NMDARs allow calcium ion influx, which acts as a second messenger and promotes synaptic strengthening or weakening by regulating the synaptic protein content and density. The initial characterization of the NMDAR interactome via MS identified a 2–3 MDa multiprotein complex demonstrating the large and complicated nature of post-synaptic protein networks. This NMDAR multi-protein complex, referred to as the hebbosome by the authors of the initial study, comprises receptors, scaffolding proteins, and secondary messenger pathway effectors thought to be key to LTP/LTD induction (Husi et al., 2000; Grant and O’Dell, 2003).

Post-synaptic kinases and phosphatases have been the focus of intense investigation for their roles in coordinating the changes in synaptic protein composition, cytoskeletal architecture, and gene expression that underlie synaptic plasticity (Thomas and Huganir, 2004; Lee, 2006; Coba, 2019). Li et al. (2016) turned the power of MS to cataloging phosphoproteomic changes after LTP, an undertaking made possible not only by the increased sensitivity and resolution of modern MS equipment, but also the wealth of bioinformatic information available to researchers (Bai et al., 2017). The authors began by identifying the kinases present at the synapse via MS analysis of the synaptic proteome and TiO2 enrichment of phosphopeptides, which identified 79 kinases representing all branches of the phylogenetic kinase tree, prompting the question: are these kinases active during LTP and what are they doing? Since kinases add phosphate groups to other proteins and are themselves often regulated by phosphorylation, a detailed inventory of changes in phosphorylation state during LTP could unearth insights into how different aspects of LTP are coordinated through cellular signaling events. Li and colleagues identified phosphorylation sites and quantified phosphopeptides by LC-MS/MS in synaptic proteins from hippocampal neurons with and without high frequency stimulation (i.e., with and without LTP). Interestingly a majority of the identified synaptic kinases were not differentially phosphorylated after LTP. This could be interpreted to mean less synaptic kinase activation during LTP than previously thought. However, the failure to detect changes in phosphorylation state in more synaptic kinases may also reflect certain technical limitations of the study. By inducing LTP via tetanic stimulation of Schaffer collateral fibers and analyzing the CA1 region of the hippocampal slice via MS, the authors introduce noise from non-neuronal cell-types and synapses in which LTP may not have been induced. However, it is notable that some of the changes in phosphorylation state of some substrates were profound enough to be detectable via western blot, suggesting activation strong enough to induce phosphorylation changes of some targets.

The authors identified proteins whose phosphorylation status was changed after LTP induction, and these represented many classes of proteins, including glutamate receptors, scaffolding proteins, and RasGAPs. Additionally, the authors predicted which class of kinase phosphorylated each phosphosite, by comparing the amino acid sequence of each phosphosite to known targets of different kinases. Their data suggest that CMCG and CAMK family kinases phosphorylate cell adhesion molecules, cytoskeletal molecules, and scaffolding molecules, whereas CAMK and AGC family molecules phosphorylate glutamate receptors, gap junction proteins, and RasGAPs. Thus, distinct signaling pathways may modulate different aspects of LTP. These findings will need to be validated with focused studies on particular kinases and their specific substrates. However, this is a good example of using phosphoproteomic screening approaches to open new avenues of investigation.

## MS in Neurological Disorder

Because complex protein interaction networks underlie important neuronal structure and function, it follows that perturbations of these networks cause neurological disorders. Many studies on neurological disorders have leveraged high throughput sequencing techniques to either look for mutations in patients’ genomes or changes in their transcriptomes. Although these techniques have yielded several key footholds, a full view of most neurological disorders remains elusive. This is, in part, because genomics and transcriptomics do not offer a full picture of disease states and can only serve as proxies when studying protein networks. Neither technique can accurately predict protein abundance or offer fine-grain information about protein localization, PTMs, or details about PPI. MS-based proteomics is capable of filling in some of these gaps. New proteomic studies on neurological disease are abundant. Here we do not offer an exhaustive review, but instead highlight studies in autism spectrum disorder (ASD), schizophrenia (SCZ), and Alzheimer’s disease (AD) that illustrate the ways in which proteomic analysis can build on genomic and transcriptomic findings, refine popular hypotheses about disease etiology, and identify new targets for treatment.

### Autism Spectrum Disorder

Autism spectrum disorder is a highly heritable, heterogeneous disorder, characterized by impairments in social communication and sensory perception, often accompanied by repetitive behaviors (Lord et al., 2018). Changes in brain connectivity have been observed in brain imaging studies with autistic participants, but ASD animal models point to a variety of possible causes, including altered excitatory to inhibitory ratio, dysregulated synaptic homeostasis, and irregular nervous system development (Mullins et al., 2016; Lord et al., 2018). As the name suggests, ASD encompasses a number of phenotypic subtypes (Lord et al., 2018). This variability is due in part to the varied genetic underpinnings of the disorder, which are thought to arise from complex inheritance involving large effect *de novo* mutations and small effect rare or common variants (Masi et al., 2017; Lord et al., 2018; Iakoucheva et al., 2019). Studies of high penetrance *de novo* gene mutations have been particularly fruitful in identifying ASD-related genes and the proteins they encode. However, the contribution of these genes to ASD is not always clear, despite their high penetrance, and often require further study to understand their role in ASD etiology. Proteomics is an ideal tool to better understand these proteins and their signaling pathways.

One such protein is Trio, a RhoGEF, in which mutations have been identified in ASD patients (Paskus et al., 2020). Interestingly, its highly homologous paralog, Kalirin, is not highly associated with ASD. In a recent study, Paskus et al. (2019, 2020) used a label-free IP-MS approach to quantify the Trio interactome alongside the interactome of its paralog protein, Kalirin, revealing differences in their interactomes that might explain why Trio, but not Kalirin, is implicated in ASD. Among the many proteins that differentially interact with Trio, Paskus and colleagues identified CRMP family proteins CRMP1-4 (also known as DPYSLs). These proteins regulate the collapse of the growth cone in response to repulsive guidance cues, play roles in modulating synaptic plasticity, and are implicated in a variety of diseases, including ASD (Wang and Strittmatter, 1996; Stroedicke et al., 2015; Moutal et al., 2019; Li et al., 2020). The Trio-CRMP interactions may indicate a role for Trio in ASD through either regulation of network connectivity via axon guidance and dendritic patterning, or modulation of synaptic strength via LTP. Both cellular processes have been implicated in ASD, demonstrating that Trio likely impacts pathways common to different ASD genetic etiologies (Broek et al., 2014; Iakoucheva et al., 2019; Ruzzo et al., 2019).

### Schizophrenia

Schizophrenia is a heterogeneous disorder characterized by a combination of positive, negative, and cognitive symptoms, which can include delusions and hallucinations (positive), social withdrawal and anhedonia (negative), and various cognitive dysfunctions (Kahn et al., 2015). Heritability accounts for 80% of SCZ risk, which is thought to be predominantly conferred by many low-penetrance common variants, with high penetrance rare variants and copy number variants also playing a role (Sullivan et al., 2003; Purcell et al., 2014; Kahn et al., 2015). Genetic studies have successfully identified many disease-associated loci, which have led to important revelations about SCZ, including the role of synaptic protein dysregulation and the immune system (Kenny et al., 2014; Ripke et al., 2014). However, a full understanding of SCZ remains elusive, despite the abundance of genetic leads available to researchers. This is, in part, because many of the identified disease-associated genetic loci confer marginal increases in risk, and so their effects may only become apparent when studied in combination with other disease-associated loci (Ripke et al., 2014; Kahn et al., 2015).

Proteomic studies based on the findings of genetic studies have been able to fill in some of the gaps. In a study by Rosato et al. (2019) cellomic screening of 41 SCZ risk genes identified in a genome wide association study via siRNA-mediated knock-down revealed three genes–Tcf4, Tbr1, Top3b–resulting in changes in synaptic morphology related to SCZ. MS-based analysis with data independent acquisition identified distinct proteomes regulated by each gene and PPI enrichment analysis indicated that the Tcf4 and Top3b knockdown proteomes were not significantly enriched for PPI, leaving it unclear which molecular pathway Tcf4 and Top3b act on. Although PPI analysis of Tbr1 identified a statistically significant PPI network, the network was small and contained only two proteins known to be dysregulated by Tbr1 knock-down. However, when the dysregulated proteins of all three knockdown conditions were analyzed in combination, PPI analysis identified a highly significant and large network containing molecules important for SNARE binding, demonstrating the importance of analyzing SCZ risk genes in concert rather than isolation. Alterations in neurotransmitter release, for which SNAREs are essential, are central to many theories of the mechanisms underlying SCZ, most notably hypotheses about the roles of glutamate, dopamine, and GABA, and thus the central finding of Rosato et al. (2019) is consistent with some current theories about SCZ (Kahn et al., 2015).

Identification of common pathways in SCZ can also be accomplished without screening candidate genes in advance. By using human inducible pluripotent stem cells (hiPSCs) from SCZ patients, researchers forgo the need to simultaneously knockdown several genes and search for the appropriate combination of risk factors to yield a disease phenotype. Use of hiPSCs also permits the study of the human PPIs, in contrast to animal models. This technology also circumvents issues in variability common to studies using post-mortem human tissue and shortcomings in verisimilitude common to animal disease models. Taking this approach, Tiihonen et al. (2019) used both transcriptomic and proteomic profiling to search for differential expression of genes in hiPSCs derived from monozygotic twins with one affected and one unaffected sibling. Proteomic analysis not only revealed commonly up- and down-regulated proteins in affected vs. unaffected individuals, but also identified a distinct dysregulated proteome in affected females compared to their unaffected counterparts. Thus, while there may be some molecular commonalities in SCZ across sex, there is a significant contribution of sex-specific molecular changes. Given the differences in SCZ presentation across patients of different sex, these results are unsurprising and warrant further investigation (Castle et al., 1998; Tandon et al., 2008; Kahn et al., 2015).

### Alzheimer’s Disease

Alzheimer’s disease is a well-studied neurological disease involving the gradual loss of synapses in older adults and is characterized by the presence of amyloid plaques and tau tangles, which are thought to play converging roles in the pathogenesis of AD. Much of the research to date has focused on genomic and transcriptomic data, which successfully identified causative genes and risk genes for AD and broadened the scope of AD research to include interactions with the immune system (Masters et al., 2015). Despite the well-known genetic underpinnings and molecular hallmarks of AD, treatment options remain limited, demonstrating that our understanding of AD is incomplete. Proteomic analyses may offer a way to fill in the details missing from popular AD models.

In a recent paper, Dejanovic et al. (2018) studied proteomic changes in a mouse model for tauopathies, which may be relevant to our understanding of AD. Dejanovic et al. (2018) identified differentially expressed synaptic proteins in Tau P301S mice (Tg) vs. wild type mice using label-free quantitative MS. GO term analysis discovered enrichment of receptors, synaptic plasticity related proteins, and synaptic transmission related proteins in the dataset of proteins downregulated in Tg mice, consistent with synaptic loss, which is a hallmark of tauopathies. Although GO term analysis indicated enrichment of metabolism-related proteins in the dataset of proteins up-regulated in Tg mice, Dejanovic et al. (2018) focused on C1q, which is upregulated at the synapse in Tg mice. C1q is a component of the complement pathway, which helps target microglia to debris for engulfment. Microglia are hypothesized to erroneously engulf and sculpt away healthy synapses in AD brains, and so the upregulation of C1q at Tg synapses suggests a mechanism by which healthy synapses are marked for destruction by microglia (Hong et al., 2016; Salter and Stevens, 2017; Hansen et al., 2018; Shi and Holtzman, 2018; Ibrahim et al., 2020). Indeed, neutralization of C1q via antibody prevents microglia engulfment of C1q and rescues synapse density in Tg mice.

It is important to note that studies in disease models come with advantages, but also important caveats. Disease models are rarely accurate representations of the disease, but rather useful tools to understand specific mechanisms of a disease. Mouse models of AD are no exception; several popular mouse models for AD exist, such as the APP/PS1 and 5 × FAD mice, and each capture different mechanistic details of the disease. Indeed, when mapped to the differentially regulated proteins identified in Dejanovic et al. (2018) several AD-related proteins are not significantly differentially expressed, indicating that the TauP301S mouse model does not fully recapitulate AD. Working with disease models in proteomic research also has important benefits, namely that sophisticated protein tagging techniques can be utilized. For example, mice expressing the mutant methionine transferase used in BONCAT under a microglia-specific promoter could be bred with Tau P301S mice to permit microglia-specific proteomic analysis, which may be instructive to our understanding of how microglia identify and attack neurons in AD. Expansion of proteomic techniques in disease research will be important to furthering our understanding of AD and other diseases.

## Limitations and Future Directions

Evidently, MS-based proteomics is a powerful tool that can be used to gain a more complete view of cellular processes, disease states, and specific sub-proteomes, and build on the knowledge gained through decades of genomic and transcriptomic research. In this review, we have curated a set of papers meant to illustrate the power of MS in neuroproteomics; however, it is important to note that this review is not exhaustive and leaves some issues unaddressed. The papers we have reviewed use bottom-up proteomics, in which proteins are digested into smaller peptides before being subjected to MS analysis. The MS and MS/MS data of these peptides are often searched against a protein database to identify sequences that best match the *in silico*-generated pattern. This approach permits identification of thousands of proteins per sample, and so has been widely adopted because peptides are easier to separate via liquid chromatography and have higher ionization efficiency compared to intact proteins. However, in bottom-up proteomics, only a portion of all peptides generated are recovered. An estimated 95% of human pre-mRNA is thought to undergo alternative splicing, and the diversity of protein isoforms is thought to play a major role in supporting the complexity of the nervous system (Pan et al., 2008; Wang et al., 2008). To recognize specific isoforms, a peptide sequence unique to the isoform must be detected; however, many isoforms are not identifiable from a unique peptide and can only be identified in their intact form (Brosch et al., 2011; Tran et al., 2011; Ezkurdia et al., 2012). Additionally, any protein isoform is necessarily lower abundance than the protein in general, which further complicates the issue of faithfully detecting protein isoforms (Heller et al., 2012). The incomplete sequence coverage in bottom-up proteomics also makes it very difficult to do in-depth PTM screening. Information about how PTMs interact with each other on an intact protein is lost. PTMs on the same protein are commonly expressed in patterns that bear biological significance. The ability to analyze these patterns is diminished when the protein is digested into smaller peptides (Duncan et al., 2010). Top-down MS, in which intact proteins are analyzed, can overcome these limitations, but come with significant technical challenges (Duncan et al., 2010). Off-line fractionation is often needed prior to on-line separation for top-down analysis, which affects the throughput of the analysis. Furthermore, detection and fragmentation of intact proteins demand high-performance mass spectrometers. The fast development in activation methods including electron transfer dissociation (ETD) and ultraviolet photodissociation (UVPD) have increased the chance of getting good sequence coverage; however, it is still difficult to accurately determine the monoisotopic mass, and therefore identify, proteins with mass larger than 50 kDa. In addition, top-down data processing and interpretation are very complicated (Catherman et al., 2014). Despite these challenges, top-down proteomics has been used to characterize full-length proteins, although typically with low-throughput (Boyne et al., 2006; Ge et al., 2009). Advances in protein fractionation techniques and ion analysis procedures have improved the throughput of top-down proteomics, making it a more viable alternative to bottom up proteomics than it has been in the past (Tran et al., 2011; Kafader et al., 2020). Until top-down techniques are widely adopted, these limitations must be acknowledged in data interpretation and resolved through alternative experimental approaches.

The field of proteomics is also inherently limited by the inability to amplify proteins. Genomic and transcriptomic analysis can derive increased sensitivity from DNA and RNA amplification techniques–namely PCR–which permits detection and sequencing of virtually all molecules. No such technique exists for proteins, and such increases in sensitivity are gained through advances in MS technology and protein enrichment schemes. Nevertheless, some protein species, though present, will remain undetectable. Even high sensitivity MS may not accurately identify some proteins. Bottom-up MS relies on peptide to protein matching, which relies on complete, well-annotated protein databases. Peptides representing proteins that are not in the database may be erroneously matched, or not matched at all (Duncan et al., 2010). Advances in technology and MS workflows may close the sensitivity gap between proteomics and nucleic acid-omics, and high-throughput approaches to *de novo* protein sequencing may eventually render database matching obsolete (Bekker-Jensen et al., 2017; Muth et al., 2018). In the meantime, researchers must be careful when interpreting their data and validate their results with orthogonal techniques.

## Conclusion

To summarize, improvements in MS experiments over the last decade have reinvigorated our efforts to understand the brain’s proteome and how it is dynamically regulated. Advances in technology have allowed researchers access to specific protein networks, insight into PTMs, and proteomic snapshots of temporally dynamic neuronal processes. The application of MS in translational research has enhanced our understanding of neurological disorders and pointed to new molecular clues in our ongoing search for therapies. Although there are still roadblocks to studying certain populations of proteins (i.e., protein isoforms) via MS, the continuing development of new techniques and improvement of existing technology promises to expand the researcher’s toolkit and eventually render the entire proteome accessible.

## Author Contributions

EF and YL wrote the manuscript. KWR and YL provided editorial guidance and supervision. All authors contributed to the article and approved the submitted version.

## Conflict of Interest

The authors declare that the research was conducted in the absence of any commercial or financial relationships that could be construed as a potential conflict of interest.
